# Genotype-environment interaction of genotypes of cocoa in Mexico

**DOI:** 10.1038/s41598-025-00162-8

**Published:** 2025-05-02

**Authors:** Carlos Hugo Avendaño-Arrazate, Misael Martínez-Bolaños, Ana Laura Reyes-Reyes, Marco Aurelio Aragón-Magadán, Delfino Reyes-López, Fernando López-Morales

**Affiliations:** 1 Instituto Nacional de Investigaciones Forestales, Agrícolas y Pecuarias, Centro Nacional de Recursos Genéticos, Av. de la Biodiversidad 400, Col. Las Cruces, Tepatitlán, Jalisco Mexico; 2 Campo Experimental Rosario Izapa, Km. 18 Carretera Tapachula-Cacahotán, C.P. 30870 Tuxtla Chico, Chiapas Mexico; 3 Facultad de Ciencias Agrícolas y Pecuarias, Benemérita Universidad Autónoma de Puebla, C.P. 73965 San Juan Acateno, Teziutlán, Puebla Mexico

**Keywords:** Genotype-environment interaction, Cacao breeding, Agronomic traits, Stability analysis, Yield performance, Plant ecology, Ecology, Genetics, Plant sciences, Ecology

## Abstract

Cacao (*Theobroma cacao L.*) production is significantly influenced by genotype-environment interactions, which affect key agronomic traits such as yield, fruit quality, and disease resistance. Despite advances in cacao breeding, one of the main challenges remains the identification of stable, high-performing clones that can adapt to different environmental conditions while maintaining desirable agronomic characteristics. In particular, the introduction of improved clones with high productivity and tolerance to diseases such as frosty pod rot is essential to ensure sustainable production. However, limited information exists on the environmental influence on these traits, making it crucial to assess the performance of new cacao clones in diverse agroecological conditions before their large-scale adoption. With the objective of evaluating the genotype-environment interaction of cacao clones generated by INIFAP and introduced for their tolerance to diseases (frosty pod rot), high yield, and quality, 23 cacao clones were established in three environments: (1) Campo Experimental Rosario Izapa-Tuxtla chico, Chiapas; (2) Ejido Umoa, Tapachula, Chiapas y (3) Vicente Guerreo, Teapa, Tabasco. The cacao clones were established in a Randomized Block Experimental Design with three replications and five trees per block. Statistical analysis was conducted in R 4.4.1 using the statgenGxE package and hierarchical clustering. The variance analysis showed highly significant differences among the genotypes for all the evaluated variables, indicating considerable genetic variability in terms of fruit length (FL), fruit weight (FW), total number of seeds (TNS), individual seed dry weight (ISDW), and pod index (PI). Environment 1, located in Rosario Izapa, Chiapas, stood out as the best for the variables FL, TNS, and ISDW. On the other hand, environment 3, situated in Vicente Guerrero, Teapa, Tabasco, proved to be the most suitable for FW and PI. In contrast, environment 2, located in Ejido Umoa, Tapachula, Chiapas, ranked as the worst for most of the variables, particularly for FL, FW, and TNS. The sensitivity analysis of the cacao genotypes depended on the type of variable; for the fruit variables, genotype 5 stood out for FL, genotype 18 for FW, genotype 4 for TNS, genotype 9 for ISDW, and genotype 15 for PI. In general, the best genotype for fruit variables was genotype 25 (F19P3). Finally, it was observed that among the 23 promising cacao genotypes, most exhibited interaction with the environment, suggesting that it is very important to consider the environmental conditions where the varieties will be established. The findings of this study provide valuable insights into the influence of environmental conditions on cacao productivity and quality, highlighting the need for site-specific clone selection. The identification of high-yielding and stable genotypes can facilitate more efficient breeding strategies and better decision-making for cacao producers and researchers. By understanding how different environments impact agronomic traits, this research contributes to the sustainable intensification of cacao cultivation, helping to optimize yields while mitigating the risks associated with environmental variability. These results are particularly relevant for breeding programs aimed at developing resilient cacao varieties suited to diverse agroecological regions.

**Supplementary Information:**

The online version contains supplementary material available at 10.1038/s41598-025-00162-8.

## Introduction

Cacao (*Theobroma cacao* L.) is a crop currently cultivated in different environments in tropical and subtropical regions across various continents due to the increasing demand for its beans, the main ingredient in chocolate production^1^. In Africa, particularly in major cacao-producing regions like Côte d’Ivoire and Ghana, genotype-environment interaction studies are being conducted before releasing improved varieties to farmers^2,3^. This is because all crops respond differently to the environments in which they are grown, and cacao, in particular, requires very specific conditions, such as agroforestry systems, which are themselves complex to manage^4^.

Several authors, agree that genotype-environment interaction studies for improved crops should be conducted in contrasting environments regarding soil type, climate, vegetation, and precipitation^5–7^. This aims to obtain the agronomic performance, growth, and development of the new genotype to be released, thereby determining its range of adaptation, whether broad or specific. Furthermore, these evaluations allow for a better understanding of how the variety will perform in each locality or region. This is because identical genotypes, as in the case of cacao when clones are grown, can exhibit different phenotypes when exposed to different environments^8,9^.

In Mexico, cacao is mainly grown in four states, with Tabasco being the most important in terms of area and production, followed by Chiapas, Oaxaca, and Guerrero^10^. However, due to the current inability to meet demand, federal government policies and private companies are expanding cacao cultivation to other states such as Veracruz, Michoacán, and Nayarit, where latitude, relative humidity, and precipitation conditions are not necessarily ideal for the crop. Therefore, before recommending cacao genotypes for these new cultivation areas, it is essential that their performance has been evaluated in different environments^11,12^. Several studies have demonstrated that crops respond positively or negatively to the environment through yield components, growth, or development^13,14^. In cacao, Quintana et al. (2015) evaluated three clones, ICS 60, ICS 95, and CCN 51, and found differences between environments in the percentage of shell and bean index. However, when assessing performance within each clone, they observed consistency in the altitude range of 200 to 1200 m in Colombia^15^. Similarly, Cheng et al. (2016), working with coffee (*Coffea arabica* L.), a crop also grown in agroforestry systems, demonstrated that some environmental factors, such as shade, positively improve coffee quality but also reduce yield^16^.

To evaluate the genotype-environment interaction of cacao clones generated by INIFAP and introduced for their tolerance to diseases (frosty pod rot), high yield, and quality, 23 cacao clones were established in three environments: two in the Soconusco region in the state of Chiapas, which is characterized by warm and semi-warm climates. The predominant climate is warm subhumid with summer rains, followed by a warm humid climate with abundant summer rainfall. The prevailing climate type is Aw2(w)ig, with an average relative humidity of 79.4% and an annual mean temperature of 26.8 °C. The predominant soil types are Andosol and Fluvisol^17^.

The third environment was located in the Sierra region of the state of Tabasco, Mexico. This region is characterized by high precipitation, averaging between 3000 and 4000 mm per year, and an average temperature of 25 °C. The predominant soil types are Vertisols, Luvisols, Leptosols, Cambisols, and Acrisols^18^.

## Materials and methods

### Cacao genotypes evaluated

A total of 23 cacao genotypes of different genetic origins were evaluated (Table 1), six of which are clones from the Tropical Agricultural Research and Higher Education Center (CATIE) in Costa Rica, and 17 were generated from the germplasm bank of the National Institute of Forestry, Agricultural and Livestock Research (INIFAP) in Mexico. These genetic materials exhibit differences in resistance or susceptibility to frosty pod rot (*Moniliophthora roreri*), as well as variations in quality and aroma.

**Table 1:** Promising cacao genotypes generated by INIFAP and clones introduced from CATIE, Costa Rica, evaluated in three environments.

ID	Clone	Characteristic	Genetic Origin
C1	CATIE R1	Resistant	Forastero
C2	CATIE R4	Resistant	Forastero
C3	CATIE R6	Resistant	Forastero
C4	CC-137	Resistant	Forastero
C5	ICS 95	Resistant	Forastero
C6	PMCT 58	Resistant	Forastero
C7	Regalo de Dios	Resistant	Trinitario
C8	Lacandón	Susceptible, Quality, Aroma	Criollo
C9	CAERI-1	Resistant	Forastero
C10	CAERI-2	Moderately tolerant, High Yield	Forastero
C11	Verde Gustavo	Susceptible, Quality	Criollo
C12	Rojo Gustavo	Susceptible, Quality	Criollo
C13	Rojo Samuel	Susceptible, Quality	Criollo
C15	Carmelo	Susceptible, Quality, Aroma	Criollo
C17	CAERI-9 (Surco 3–4)	Susceptible, Good Yield	Forastero
C18	CAERI-5 (T8-28)	Susceptible, Yield	Forastero
C19	SCA-6	Resistant	Forasterp
C20	SCA-12	Resistant	Forastero
C21	PORCELANA	Susceptible, Quality, Aroma	Criollo
C22	CAER-6 (I-111)	Susceptible, Quality	Trinitario
C23	CAERI-7 (I-161)	Susceptible, Quality	Trinitario
C24	CAERI-8 (F10-2)	Resistant, Quality	Trinitario
C25	CAERI-4 (F19-3)	Tolerant, Quality, Yield	Trinitario

All materials are preserved in the National Cacao Germplasm Bank at the Rosario Izapa Experimental Field, located in Tuxtla Chico, Chiapas, Mexico, with identification numbers ranging from C1 to C25 (Table 1). These materials are part of the national cacao genetic improvement program of INIFAP, Mexico, and have been generated or evaluated within this program.

### Evaluation environments

The cacao genotypes were evaluated in three environments:

Environment 1: Rosario Izapa Experimental Field, municipality of Tuxtla Chico, Chiapas, located at 14° 58′ 18″ N and 92° 09′ 14″ W, at an altitude of 443 m, with an annual precipitation of 3204 mm, maximum temperature of 32.4 °C, and minimum temperature of 19.5 °C.

Environment 2: Ejido Umoa, municipality of Tapachula, Chiapas, located at 14° 49′ 52″ N and 92° 13′ 51″ W, at an altitude of 97 m, with an annual precipitation of 1389 mm, maximum temperature of 35.1 °C, and minimum temperature of 22 °C.

Environment 3: Vicente Guerrero, Teapa, Tabasco, located at 17° 30′ 37″ N and 92° 55′ 04″ W, at an altitude of 95 m, with an annual precipitation of 3242 mm, maximum temperature of 31.4 °C, and minimum temperature of 21 °C.

### Evaluated variables

Five variables related to yield components, which are considered by producers for the selection of cacao clones, were evaluated. These variables were measured in each harvest cycle in 15 trees per clone and per environment, all of them five years old:

Fruit Length (FL, cm), Fruit Weight (FW, g), Total Number of Seeds (TNS), Individual Seed Dry Weight (ISDW, g), and Pod Index (PI) (Number of fruits required to produce one kilogram of dry seeds). After harvesting the fruits, they were transported to the laboratory for analysis. Twenty fruits per tree were selected, and the total and individual seed weight, as well as the number of seeds per fruit, were recorded. The dry seed weight was determined at 7% moisture. Data were collected twice a year (in each harvest cycle) over a period of two consecutive years.

### Experimental design

The treatments corresponded to the 23 cacao clones, distributed in a randomized complete block design with three replications. Each replication consisted of five cacao plants obtained by grafting.

### Agronomic management

The agronomic management of the experimental plots in the three environments was carried out following the technological production package proposed by INIFAP^19^. The plots were established under temporary shade (*Cajanus cajan*) and permanent shade (*Gliricidia sepium*), with a planting distance of 3 m between plants and rows. Each plant received an annual application of 900 g of triple 17 fertilizer (17N-17P-17K), distributed in three applications every four months. Weed control was performed manually.

During the first six months of plant growth, formative pruning was conducted, followed by maintenance and fruiting pruning. Throughout the experiment, diseased fruits were regularly removed, and copper oxychloride treatments were applied at a dose of 3 g/L of water. Harvesting was performed manually.

### Statistical analysis

The data were organized in a table in CSV format (Supplementary Material 1), and all statistical analyses and genotype-environment interaction analyses were performed using the R statistical software^20^, version 4.4.1. To reduce noise that could affect the analysis and interpretation of the results, data preprocessing was conducted by removing outliers. These outliers were identified using the Interquartile Range (IQR) method in R, considering as outliers those values falling outside the range defined by Q1 − 1.5(IQR) and Q3 + 1.5(IQR).

Subsequently, a variance analysis was performed according to the Finlay-Wilkinson method for each variable. The Finlay-Wilkinson analysis^21^ describes the genotype-environment interaction through the heterogeneity of the slopes of a regression of individual genotypic performance on an environmental index. The environmental index is the average of all genotypes in a particular environment. The intercept represents the overall performance across all environments, the slope represents adaptability, and the residuals may indicate a measure of stability.

### Stability measures analysis

Stability measures were calculated using the statgenGxE package^22^ to assess the genotype × environment (GxE) interaction, the cultivar superiority measure of Lin and Binns, Shukla’s stability variance, and Wricke’s ecovalence. The superiority of a cacao genotype is a function of the sum of the squared differences between a cultivar’s mean and the mean of the best cultivar, where the sum is across trials. Genotypes with smaller superiority values tend to be more stable and closer to the best genotype in each environment. Shukla’s stability variance (static stability) is defined as the variance around the phenotypic mean of the genotype across all environments. This provides a measure of genotype consistency, regardless of yield. Wricke’s Ecovalence Stability Coefficient measures each genotype’s contribution to the GxE sum of squares in an unweighted analysis of GxE means. A low value indicates that the genotype responds consistently to environmental changes, i.e., it is stable from a dynamic perspective.

Additionally, a principal component analysis and hierarchical cluster analysis was performed to compare the variables, environments, and cacao genotypes.

## Results and discussion

### Genotype × environment interaction analysis of 23 cacao genotypes

The mean squares from the variance analysis (Table 2) following the Finlay-Wilkinson (1963) model indicate significant differences in fruit and seed variables among environments, genotypes, and genotype × environment interactions, except for the variable Total Number of Seeds (NST) among environments.

**Table 2:** Mean squares for fruit and seed variables of 23 cacao genotypes evaluated in three environments.

Source	DF	FL	FW	NST	ISDW	PI
Genotypes	22	100.16***	767,431***	710.4***	1.07***	2378.6***
Environments	2	213.73***	2,862,599***	150.8ns	0.13*	458.9*
Env*Gen	43	7.49***	99,394***	88.0*	0.23***	381.6***
Residual	885,475	381.00	32,606	55.7	0.04	134.6

**Highly significant;*Significant; ns: not significant. FL: Fruit Length; FW: Fruit Weight; NST: Total Number of Seeds; ISDW: Individual Seed Dry Weight; PI: Pod Index.

The variance analysis of fruit and seed variables showed highly significant differences among genotypes for all evaluated variables, indicating considerable genetic variability in fruit length (FL), fruit weight (FW), total number of seeds (NST), individual seed dry weight (ISDW), and pod index (PI). These results suggest the presence of genotypes with superior potential in some of these traits, which is important for selecting improved varieties.

On the other hand, environmental effects were also significant for most of the evaluated variables, except for NST, which did not show significant differences between environments. This suggests that the total number of seeds is a more stable trait, less affected by environmental conditions, whereas the other variables are more influenced by environmental variations.

The genotype × environment (GxE) interaction was significant for all variables, indicating that genotypes respond differently depending on the environment in which they are grown. This highlights the importance of evaluating both the stability and adaptability of each genotype under different environmental conditions before recommending them for new cultivation areas. In particular, the GxE interaction suggests that some genotypes may perform well in certain environments but not necessarily in others.

Similar results were reported by Ofori et al. (2023), who evaluated 23 cacao hybrids in different environments and found highly significant differences in the mean squares for environments, hybrids, and their interaction between environments and cacao hybrids.

Finally, the analysis revealed significant residual variance, indicating the possible influence of uncontrolled factors in the experiment, such as agricultural management practices, microenvironmental variations, or genetic differences not considered in the analysis.

The analysis of the environmental effects on the evaluated variables for the 23 cacao genotypes revealed significant differences between the three studied environments (Table 3). Environment 1, located in Rosario Izapa, Chiapas, stood out as the best for fruit length (FL), total number of seeds (NST), and individual seed dry weight (ISDW). This suggests that the conditions of this environment, possibly related to climate and soil, are particularly favorable for the development of longer fruits with a greater number of seeds and higher seed weight.

**Table 3:** Mean squares for the environmental effects on 23 cacao genotypes evaluated in three environments.

Environment	FL	FW	NST	ISDW	PI
1	529,734.4*	− 17.66	1.51*	0.05*	− 1.29**
2	− 264,867.2**	− 106.83**	− 0.92**	− 0.02	− 2.79
3	− 264,867.1	124.50*	− 0.59	− 0.03**	4.09*

*Best environment; **Worst environment. FL: Fruit Length; FW: Fruit Weight; NST: Total Number of Seeds; ISDW: Individual Seed Dry Weight; PI: Pod Index.

On the other hand, environment 3, located in Vicente Guerrero, Teapa, Tabasco, proved to be the most suitable for fruit weight (FW) and pod index (PI). This environment seems to favor the production of heavier fruits and a lower number of fruits required to produce one kilogram of dry seeds, which is advantageous from a production standpoint. However, this environment was not as favorable for other key variables, such as seed dry weight and the number of seeds.

In contrast, environment 2, located in Ejido Umoa, Tapachula, Chiapas, was ranked as the worst for most variables, particularly for fruit length, fruit weight, and total number of seeds. These observations suggest that the conditions in this environment are suboptimal for cacao cultivation, which could be related to factors such as lower precipitation or differences in altitude, negatively affecting fruit yield and development.

These results emphasize the importance of considering the genotype × environment interaction when selecting cultivation areas. Different environments favor distinct yield variables, implying that the choice of environment should align with production objectives, whether prioritizing larger fruits, heavier weight, or better grain quality. Arunkumar et al*.* (2019) mention that when evaluating cacao trees for flowering, development, and yield, fruit weight traits were variable and highly influenced by genetic and environmental factors, including soil moisture and nutritional status^23^. They also mention that for selecting promising cacao genotypes, traits such as individual seed dry weight, yield, and dry seed weight per tree are important. In this regard, Agudelo-Castañeda et al*.* (2023) found that when evaluating eight cacao genotypes in different environments, the most important variables were yield and the number of pods per tree. Bekele et al. (2020) further note that when selecting cacao clones, pod indices below 21 to 25 are preferred, as this allows for selecting trees that produce heavier and larger seeds^24^.

Overall, the identification of highly significant differences among genotypes for all evaluated variables suggests considerable genetic variability within the studied population. The significant environmental effects on most variables, except for the total number of seeds, indicate that edaphoclimatic conditions may have a substantial influence on cacao yield and quality. Specifically, optimal environments were identified for different variables of interest, with Rosario Izapa, Chiapas, emerging as the most favorable environment for the development of longer fruits with a higher number of seeds, while Vicente Guerrero, Tabasco, promoted the production of heavier fruits with greater dry weight efficiency per fruit. These findings underscore the need to tailor genotype selection to the specific conditions of each production area to maximize fruit productivity and quality.

### Sensitivity and stability analysis of 23 cacao genotypes

The sensitivity analysis (Finlay and Wilkinson) of cacao genotypes depended on the type of variable (Table 4). For fruit variables, genotype 5 stood out for Fruit Length (FL); genotype 18 for Fruit Weight (FW); genotype 4 for Total Number of Seeds (NST); genotype 9 for Individual Seed Dry Weight (ISDW); and genotype 15 for Pod Index (PI). Overall, different behaviors were observed among genotypes and variables.

**Table 4:** Sensitivity analysis of 23 cacao genotypes evaluated in three environments.

LF		FW		NST		ISDW		PI	
Gen	Avg	Gen	Avg	Gen	Avg	Gen	Avg	Gen	Avg
5	2.2	18	2.7	4	9.9	9	15.2	15	17.1
7	2.0	5	2.5	11	7.1	21	7.7	9	4.7
18	1.9	7	1.8	19	3.6	25	6.5	13	4.4
6	1.6	3	1.7	2	3.0	10	4.9	23	3.5
22	1.5	6	1.6	25	3.0	5	3.4	2	3.3

The sensitivity analysis of the 23 cacao genotypes, based on the Finlay-Wilkinson regression model, revealed significant differences in the adaptability of the genotypes evaluated in three environments. For fruit length (FL), genotype 5 showed the highest sensitivity (2.2), indicating that this genotype has a high capacity to adapt to favorable environmental conditions. Other genotypes, such as genotypes 7 and 18, also exhibited significant responses to environmental changes, while genotype 22 demonstrated lower sensitivity (1.5), indicating that it is more stable across different environments.

For fruit weight (FW), genotype 18 was the most sensitive (2.7), responding very favorably in optimal environments. However, this high sensitivity also implies greater susceptibility to fluctuations in less favorable environments. Genotypes 5 and 6 showed lower sensitivities, making them more stable across a wider range of environmental conditions.

Regarding the total number of seeds (NST), genotype 4 stood out with high sensitivity (9.9), indicating great variability in its performance based on the environment. This behavior was similar to that of genotype 11 (7.1), suggesting that both genotypes are suitable for favorable environments but may not be as consistent in more adverse conditions.

For individual seed dry weight (ISDW), genotype 9 was the most sensitive (15.2), reflecting its dependence on optimal conditions to maximize this variable. Other genotypes, such as 21 and 25, also showed considerable sensitivity, suggesting that these genotypes are more suitable for specific environments. According to Bekele et al*.* (2022), cultivated species generally have larger fruits or seeds compared to their wild ancestors, indicating that fruit and seed size are important agronomic traits selected during crop domestication.

Finally, the pod index (PI) was most sensitive in genotype 15 (17.1), indicating that it requires favorable conditions to optimize its efficiency in dry seed production. In contrast, genotype 2 showed lower sensitivity (3.3), suggesting that it is more stable across different environments and maintains a consistent yield.

In general, genotypes with high sensitivity, such as genotypes 5, 18, 4, 9, and 15, are more suitable for cultivation in favorable environments where they can express their maximum potential. However, their performance may be more variable under adverse conditions. On the other hand, genotypes with low sensitivity, such as genotypes 6, 3, 22, and 2, are more stable and exhibit more performance that is consistent across different environments, making them more versatile for cultivation in diverse conditions. This sensitivity analysis is fundamental for genotype selection as it allows identifying those most suited for specific environments or for conditions that are more diverse.

The stability analysis of cacao genotypes using the StatgenGxA package revealed relevant results for different variables. For fruit length, genotype 4 stood out with the best superiority measure, while genotypes 13 and 25 demonstrated the greatest stability according to static stability and Wricke’s ecovalence metrics, respectively (Table 5). For fruit weight, genotype 19 showed the best superiority, indicating superior performance, although genotype 25 was the most stable in both stability metrics (static stability and Wricke’s ecovalence), suggesting its consistency across different environments (Table 6).

**Table 5:** Stability measures for the variable fruit length of 23 cacao genotypes.

Genotype	Superiority	Genotype	Static stability	Genotype	Wricke’s ecovalence
4	65.0	13	8.8	13	10.1
19	60.2	25	7.0	25	7.3
20	59.3	17	5.9	17	6.8

**Table 6:** Stability measures for the variable fruit weight of 23 cacao genotypes.

Genotype	Superiority	Genotype	Static stability	Genotype	Wricke’s ecovalence
19	961,477.1	25	146,589.6	25	173,145.4
20	438,857.3	13	94,587.1	13	99,632.0
7	743,832.0	17	71,237.4	17	74,834.8

For the total number of seeds per fruit, genotype 18 was the most outstanding in terms of superiority, but genotypes 20 and 13 showed the greatest stability, indicating that they are more reliable across a wider range of environmental conditions (Table 7). For individual seed dry weight, genotype 20 was the best in terms of superiority, but genotype 19 stood out again for its stability, maintaining consistent performance across different environments. Similarly, genotype 13 showed stability both in the seed weight variable and in others (Table 8).

**Table 7:** Stability measures for the variable total number of seeds per fruit of 23 cacao genotypes.

Genotype	Superiority	Genotype	Static stability	Genotype	Wricke’s ecovalence
18	422.9	20	43.6	13	92.7
1	427.4	13	39.8	20	76.9
19	383.5	1	24.6	19	51.6

**Table 8:** Stability measures for the variable individual seed dry weight of 23 cacao genotypes.

Genotype	Superiority	Genotype	Static stability	Genotype	Wricke’s ecovalence
20	0.39	19	0.08	13	0.14
24	0.32	13	0.05	19	0.12
19	0.30	17	0.03	17	0.10

Regarding the pod index, genotype 25 showed the greatest superiority, while genotypes 19 and 11 were the most stable, according to static stability and Wricke’s ecovalence metrics (Table 9). These results suggest that, in general, some genotypes, such as 25 and 19, present high yields in favorable conditions, but others, such as 13 and 17, stand out for their stability, making them more suitable for cultivation in variable or diverse conditions.

**Table 9:** Stability measures for the variable pod index of 23 cacao genotypes.

Genotype	Superiority	Genotype	Static stability	Genotype	Wricke’s ecovalence
25	4106.8	19	213.2	11	436.8
13	4083.5	11	192.1	19	323.6
15	4062.8	20	98.0	21	274.9

Sánchez-Mora et al*.* (2014) mention that the pod index is important for cacao clone selection and seed indices are key for annual yield and breeding studies, as the industry prefers seeds over 1.0 g. Seeds weighing less than one gram tend to have less fat and more husk.

### Principal component analysis of 23 cacao genotypes

According to the Principal Component Analysis (PCA), the first two components explain 77% of the variance for all the analyzed variables. The vector corresponding to the Total Number of Seeds (NST) is the best represented in the analysis, followed by the Pod Index (PI), Fruit Length (FL), Fresh Seed Weight (FW), and Individual Seed Dry Weight (ISDW).

**Fig. 1:**
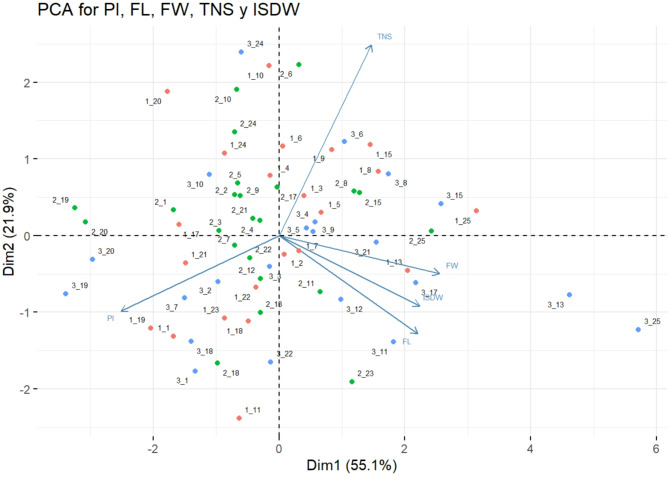
PCA plot of 23 cacao genotypes according to the variables LF, PF, NST, ISDW, and PI evaluated in three environments.

The variables FW, ISDW, and FL are strongly correlated with each other, as indicated in Fig. 1 by forming acute angles (less than 90°), and they contribute significantly and positively to the variance of the first principal component (PC1). However, these variables contribute negatively to the second principal component (PC2), suggesting that as the values of FW, ISDW, and FL increase the value of PC2 decreases.

Figure 1 also reveals that the variables FW, ISDW, and FL are not related to NST and PI, as they form angles close to 90°, indicating a low correlation. This result is consistent with expectations, given that the total number of seeds and the pod index do not depend on the weight or length of the fruit.

Additionally, the variables NST and PI are negatively related to each other, as they form an angle close to 180° between their vectors. This means that as the total number of seeds increases, the pod index (PI) decreases. This result is also consistent with expectations, as a higher number of seeds per fruit implies that fewer fruits are needed to produce one kilogram of cacao, a key characteristic in clone selection during genetic improvement^24^.

Finally, the points corresponding to environments and genotypes in Fig. 1 show a homogeneous distribution in space, without forming apparent groups, suggesting heterogeneity among the genotypes in the three evaluated environments. Exceptions include genotypes 13 and 25 in environment 3, which are grouped in the fourth quadrant of the graph, and genotypes 19 and 20 in environments 2 and 3, which are also grouped. This suggests that these genotypes share similar traits and are alike in terms of their variable expressions.

**Fig. 2:**
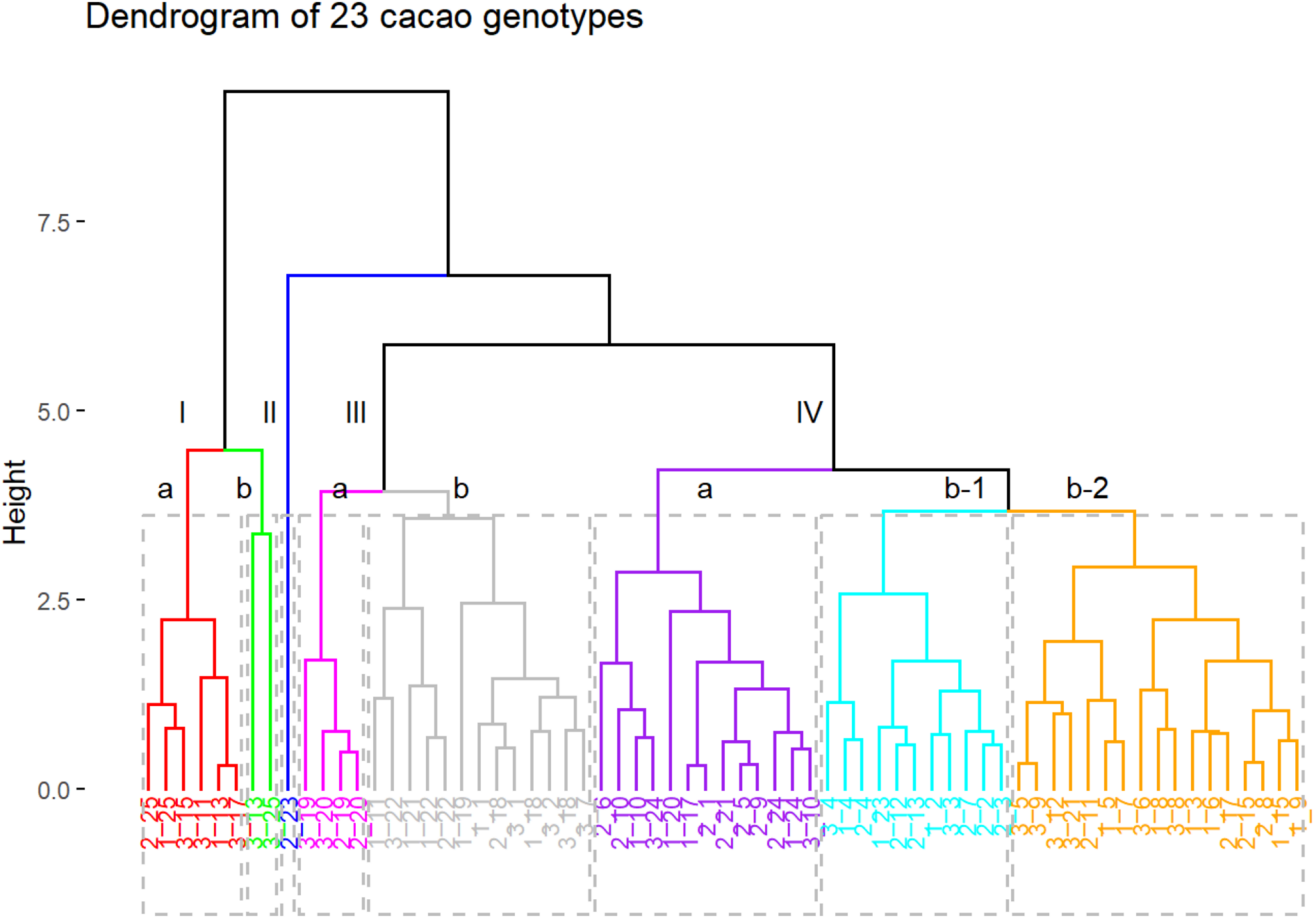
Dendrogram of 23 cacao genotypes based on the variables FL, FW, NST, ISDW, and PI evaluated in three environments.

The hierarchical cluster analysis was performed based on fruit size and weight variables, which directly impact the number and weight of seeds and, consequently, the pod index—a key characteristic for producers when selecting a variety (Fig. 2). The analysis identified four main clusters, composed of eight subgroups.

Cluster I was divided into two subgroups. Subgroup included genotypes 25, 15, 11, 13, and 17, with averages of FL of 21.35 cm, FW of 966.45 g, NST of 35.8, ISDW of 1.27 g, and a PI of 23.4. Notably, genotype 25 exhibited similar behavior in environments 1 and 2, while the rest of the genotypes in the subgroup performed more homogeneously in environment 3. Subgroup Ib consisted only of genotypes 13 and 25, which shared similar behavior in environment 3, with averages of FL of 24.6 cm, FW of 1369.4 g, NST of 38.6, ISDW of 1.73 g, and a PI of 15.56. This cluster highlights the stability of genotype 25, which showed consistent behavior across all three analyzed environments.

Cluster II consisted solely of genotype 23 in environment 2, with FL of 17.8 cm, FW of 497.6 g, NST of 23.8, ISDW of 1.93 g, and a PI of 23.8. This suggests that the performance of genotype 23 in environment 2 was notably different from that observed in environments 1 and 3.

Cluster III was subdivided into two subgroups. Subgroup IIIa included genotypes 19 and 20 from environments 2 and 3, with averages of FL of 15.5 cm, FW of 354 g, NST of 29.6, ISDW of 0.64 g, and a PI of 59.8, being the group with the smallest fruits. This subgroup suggests that these genotypes have similar yields and homogeneous behavior in environments 2 and 3. Subgroup IIIb comprised 13 genotypes, with genotypes 22 and 18 standing out for their stable behavior across the three environments, with averages of FL of 18.5 cm, FW of 527.13 g, NST of 26.5, ISDW of 1.0 g, and a PI of 43.8.

Cluster IV was subdivided into three subgroups. Subgroup IVa grouped 13 genotypes, with genotype 10 standing out for its similar behavior across the three environments, with averages of FL of 16.2 cm, FW of 453.1 g, NST of 36.8, ISDW of 0.88 g, and a PI of 34.1. Subgroup IVb1 included 10 genotypes, with genotype 4 standing out for its consistent yield across all environments, with an average of FL of 16.5 cm, FW of 467.02 g, NST of 30.2, ISDW of 1.2 g, and a PI of 29.9. Finally, subgroup IVb2 comprised 16 genotypes, being the largest group in terms of the number of genotypes across the three environments. In this subgroup, genotypes 5 and 8 exhibited similar behavior to each other and across the three environments, with averages of FL of 18.6 cm, FW of 634.9 g, NST of 35.5, ISDW of 1.17 g, and a PI of 26.07 (Fig. 2).

## Conclusions

This study analyzed the genotype × environment (G × E) interaction of 23 cacao genotypes across three distinct environments, providing insights into their adaptability and stability. The variance analysis revealed significant genetic variability among genotypes for all evaluated traits, highlighting the potential for selecting superior individuals for fruit and seed production. The environmental effects were also significant for most traits, except for the total number of seeds (NST), indicating its relative stability across environments.

The significant G × E interaction underscores the importance of evaluating genotypes under different environmental conditions before recommending them for commercial cultivation. Some genotypes exhibited superior performance in specific environments, suggesting that environmental factors such as climate and soil conditions play a crucial role in cacao production. For example, Environment 1 (Rosario Izapa, Chiapas) favored fruit length, total number of seeds, and individual seed dry weight, whereas Environment 3 (Vicente Guerrero, Tabasco) was optimal for fruit weight and pod index. Conversely, Environment 2 (Ejido Umoa, Chiapas) showed the least favorable conditions for cacao production.

The sensitivity and stability analyses provided further insights into genotype performance. High-sensitivity genotypes, such as genotypes 5, 18, 4, 9, and 15, demonstrated greater adaptability to favorable conditions but may be less stable under suboptimal environments. In contrast, genotypes with lower sensitivity, including genotypes 6, 3, 22, and 2, exhibited more stable performance across different environmental conditions. Stability analyses identified genotypes 25, 19, and 13 as the most stable across multiple traits, making them promising candidates for breeding programs targeting diverse environmental conditions.

Overall, these findings highlight the need for environment-specific genotype selection strategies to optimize cacao productivity. Future research should focus on long-term evaluations of these genotypes across additional environmental gradients and incorporate molecular analyses to further elucidate the genetic basis of their adaptability and stability.

It was observed that most of the 23 promising cacao genotypes exhibited genotype-environment interaction, indicating the importance of considering environmental conditions when establishing varieties. However, some genotypes demonstrated stability and environmental sensitivity, with genotype 25 (F19P3) standing out in fruit-related variables. This genotype was subsequently registered with the National Seed Inspection and Certification Service (SNICS) under the denomination CAERI 4.

## Electronic supplementary material

Below is the link to the electronic supplementary material.

Supplementary Material 1

## Data Availability

The datasets used and/or analysed during the current study available from the corresponding author on reasonable request.
